# Characterization of Nitrite Degradation by *Lactobacillus casei* subsp. *rhamnosus* LCR 6013

**DOI:** 10.1371/journal.pone.0093308

**Published:** 2014-04-22

**Authors:** Dong-mei Liu, Pan Wang, Xin-yue Zhang, Xi-lin Xu, Hui Wu, Li Li

**Affiliations:** College of Light Industry and Food Science, South China University of Technology, Guangzhou, Guangdong, China; National Center for Biotechnology Information (NCBI), United States of America

## Abstract

Nitrites are potential carcinogens. Therefore, limiting nitrites in food is critically important for food safety. The nitrite degradation capacity of *Lactobacillus casei* subsp. *rhamnosus* LCR 6013 was investigated in pickle fermentation. After LCR 6013 fermentation for 120 h at 37°C, the nitrite concentration in the fermentation system was significantly lower than that in the control sample without the LCR 6013 strain. The effects of NaCl and Vc on nitrite degradation by LCR 6013 in the De Man, Rogosa and Sharpe (MRS) medium were also investigated. The highest nitrite degradations, 9.29 mg/L and 9.89 mg/L, were observed when NaCl and Vc concentrations were 0.75% and 0.02%, respectively in the MRS medium, which was significantly higher than the control group (*p ≤ 0.01*). Electron capture/gas chromatography and indophenol blue staining were used to study the nitrite degradation pathway of LCR 6013. The nitrite degradation products contained N_2_O, but no NH_4_^+^The LCR 6013 strain completely degraded all NaNO_2_ (50.00 mg/L) after 16 h of fermentation. The enzyme activity of NiR in the periplasmic space was 2.5 times of that in the cytoplasm. Our results demonstrated that *L. casei* subsp. *rhamnosus* LCR 6013 can effectively degrade nitrites in both the pickle fermentation system and in MRS medium by NiR. Nitrites are degraded by the LCR 6013 strain, likely via the nitrate respiration pathway (NO_2_^−^>NO^−^>N_2_O^−^>N_2_), rather than the aammonium formation pathway (dissimilatory nitrate reduction to ammonium, DNRA), because the degradation products contain N_2_O, but not NH_4_^+^.

## Introduction

Nitrites are potentially strong carcinogens and are generated in the process of vegetable fermentation, which poses a potential food safety issue. Intake of large amounts of nitrites causes methemoglobinemia and acute poisoning [1], [2]. Under suitable conditions, nitrites react with amines, products of protein decomposition, to generate N-nitroso compounds. Over 100 N-nitroso compounds have been synthesized, so far, 80% of which are strong carcinogenins in animals [3]. However, nitrites are also widely used in the meat industry to prevent *Clostridium botulinum* growth and are also used as coloring agents. Therefore, limiting nitrites in food is highlighted in food safety research. Hashimoto *et al.* have found that inoculation of *Lactobacillus* could inhibit accumulation of high concentrations of nitrites in kimchi fermentation [4]. It has also been reported that inoculation of over 10 different strains of *Lactobacillus* bacteria, including *Lactobacillus brevis* [5], *Lactobacillus fermentum* [6], *Lactobacillus acidophilus*, and *Lactobacillus plantrum* [6], can efficiently reduce the concentration of nitrites generated by fermentation or artificially added during the fermentation of pickles and meat. Our previous study showed that inoculation of *LCR 6013* dramatically reduced the concentration of nitrites in fermented pickles [7]. However, the pathway and mechanism of nitrite degradation by the *Lactobacillus* bacteria and the key enzyme involved in this process, as well as the subcellular localization of the enzyme, are poorly understood. In this study, the role of LCR 6013 in the nitrite degradation during pickle fermentation and factors affecting nitrite degradation efficiency were investigated. In addition, electron capture/gas chromatography and indophenol blue staining were used to study the nitrite degradation pathway of LCR 6013. The enzyme activity of NiR in different cellular compartments was also examined to determine the subcellular location of NiR.

## Materials and Methods

### Bacterial Strains and Reagents

*Lactobacillus casei* subsp. *rhamnosus* LCR 6013 was preserved in the Food Safety and Inspection Center, Light Industry and Food Science College, South China University of Science and Technology. The main reagents used in this study included trypsin inhibitor (Guangzhou Qiyun Ltd., Guangzhou, China), lysozyme (Beijing Tianenze Biotech company, Beijing, China), sodium nitrite (Tianjin Fucheng Chemical Reagent, Tianjin, China), MRS medium (Guangdong Huankai Microbial Technology Co., Guangzhou, China), Vc (Tianjin Fucheng Chemical Reagent, Tianjin, China), NaCl (Tianjin Fucheng Chemical Reagent, Tianjin, China), dithiothreitol DTT (Shanghai Sigma company, Shanghai, China), and bovine serum albumin (Guangzhou Qiyun Ltd., Guangzhou, China).

### Preparation of the Solution and Medium

To prepare the PBS buffer (0.01 mol/L, pH = 7.4), NaCl (8 g), KCl (0.2 g), NaHPO_4_ (1.44 g), and KH_2_PO_4_ (0.24 g) were dissolved in 800 ml distilled water and the pH was adjusted to 7.4 with hydrochloric acid, whereupon the final volume was adjusted to 1000 ml. Next, 0.5 ml DTT (1 mol/L) and 0.2 ml trypsin inhibitor (2 µg/ml) were added to 100 ml PBS (0.01 mol/L, pH 7.4) and the final concentration was adjusted to 5 mmol/L, for preparation of the NiR enzyme extract buffer. 3.8158 g NH_4_Cl were then dissolved in 1000 ml ultrapure water to prepare the total ammonia nitrogen (TAN) stock solution (1000 mg/L). To prepare liquid MRS medium, 54 g MRS solid powder was added to 1000 mL of distilled water and mixed completely and the pH was adjusted to 6.8, using sodium hydroxide. The solution was sterilized under 0.1 MPa for 20 min by vertical pressure steam sterilizer (Shanghai Boxun Company, Shanghai, China).

### Vegetable Fermentation

One loop of LCR 6013 was inoculated into the sterilized MRS medium [3] and incubated at 37°C for 24 h, without shaking, to produce the mother starter. Then, 5% mother starter was added to liquid MRS medium to allow bacteria to reach 10^7^–10^8^ cfu/ml and then incubated at 37°C for 24 h, without shaking, to produce the bulk starter. The vegetable fermentation process was conducted according to following protocol: fresh mustard -> cleaning and drying -> segmentation and blanching -> adding 5% bulk starter -> fermentation at 37°C for 120 h in a sealed bottle -> fermented vegetable samples were regularly collected under sterile conditions to test the concentration of nitrites. The fermentation was also conducted under sterile conditions. To obtain the best growth and anaerobic fermentation of *Lactobacillus*, the vegetables were kept in plenty of bulk starter and not exposed to air. The control group (CK0) was conventional vegetable fermentation, without inoculation of LCR 6013.

### Measurement of the Nitrite Concentration in Fermented Vegetables

The fermented vegetable samples were collected after fermentation times of 0 h, 24 h, 48 h, 72 h, 96 h and 120 h, chopped, and filtrated with a 200 mesh filter cloth. One milliliter of the filtration was diluted to 100 ml using double distilled water. The nitrite concentration was determined using the GB/T5009.33-2003 method. The nitrite concentration is shown in units of mg/L. Each test was repeated three times. To measure the nitrite concentration in the MRS medium, 1 ml liquid MRS medium was diluted to 100 ml using double distilled water. Each test was repeated three times.

### Evaluation of Factors Affecting the Nitrite Degradation of LCR 6013

1. **Influence of NaCl on the nitrite degradation of LCR 6013.** First, 5% mother starter was added to liquid MRS medium containing 0.00%, 0.25%, 0.50%, 0.75%, 0.100%, 1.00%, and 1.25% NaCl, respectively. Then, 100.00 mg/L NaNO_2_ was added to the mix to reach a concentration of 10.00 mg/L. The mixture was fermented at 37°C for 24 h, without shaking. The NaNO_2_ concentration was determined according the protocol in section 1.5 and 10.00 mg/LNaNO_2_ was removed_,_ to obtain the degradation of nitrites. Each experiment was repeated three times.
2. **Influence of Vc on nitrite degradation of LCR 6013.** First, 5% mother starter of LCR 6013 was added to MRS liquid medium containing 0.00, 0.02%, 0.04%, 0.06%, 0.08%, and 0.10% Vc, respectively. Then, 100.00 mg/L NaNO_2_ was added to the mix to reach a final concentration of 10.00 mg/L. The mixture was fermented at 37°C for 24 h, without shaking. The NaNO_2_ concentration was determined according the protocol in section 1.5 and 10.00 mg/LNaNO_2_ was removed to obtain the degradation of nitrites. Each experiment was repeated three times.

### Determination of the Nitrite Degradation Pathway of LCR 6013

1. **Nitrite degradation in a closed fermentation system.** Ten milliliters liquid MRS medium containing 10.00 mg/LNaNO_2_, 0.75% NaCl, and 0.02% Vc were added to a 25 ml headspace vial. Then, 5% mother starter was added to initiate the fermentation at 37°C for 24 h, without shaking. The gas in the headspace was collected from the closed headspace vial and the N_2_O concentration was determined. The experiment was repeated three times. For control groups CK1 and CK2, the fermentation system was the same as the experimental group, but without the inoculation of LCR 6013 and without the addition of 10.00 g/LNaNO_2_, respectively.
2. **ECD- gas chromatography to detect the nitrite degradation product N_2_O.** The external standard method and 7890-II gas chromatograph (Shanghai TianMei scientific instrument Ltd. Shanghai, China) were used to measure N_2_O concentration. The concentration of standard N_2_O is 99.999%. The different concentrations of standard gas were prepared based on volume ratio. The standard curve equation was Y = (X +3500.4)/4135.6, where Y is the N_2_O concentration, X is the peak area, and the regression coefficient is R^2^ = 0.9991. The analytic conditions are as follows: column temperature was 45°C, injector temperature was 130°C, and the detector temperature was 280°C. The gas (1 ml) was injected using a syringe. Carrier gas was high-purity nitrogen with a flow rate of 30 ml/min.
3. **Measurement of Total Ammonia Nitrogen (TAN).** The concentration of TAN was measured by the indophenol blue staining method, according to our previous report [8]. Our protocol is slightly different from conventional methods for the detection of TAN. We used phenolate, rather than sodium salicylate, to reduce the accident risk. The absorbance values of 640 nm at nine points, ranging from 0 to 1.0 mg/L, were measured to generate the standard curve to detect the NH_4_^+^ concentration.
4. **The influence of fermentation time on N_2_O production.** In the above 1.6 (1) reaction system, 50.00 g/L NaNO_2_ and 5% mother starter were added to the MRS liquid medium to start fermentation at 37°C for 2 h, 4 h, 6 h, 8 h, 10 h, 12 h, 14 h, 16 h, 18 h, 20 h, 22 h, 24 h, and 26 h. Then, 1 ml of the headspace gas was collected and the N_2_O concentration was measured with three repeats.

### Determination of the Subcellular Location of NiR in LCR 6013

1. **Preparation of LCR 6013 cells containing NiR induced by nitrites.** First, 5% mother starter was inoculated to 1 L liquid MRS medium containing 5.00 g/L NaNO_2_ and fermentation was started at 37°C for 24 h, without shaking, to generate a cell broth induced by NiR (CBINR). The CBINR was centrifuged at 4°C and 8000 r/min for 30 min by 5804R desktop refrigerated centrifuge (Eppendorf, Hamburg, Germany). The cell pellet was washed twice at 4°C with pre-cooled PBS buffer (0.01 mol/L, pH 7.4) and centrifuged to prepare cells induced by nitrites reductase (CINR).
2. **Preparation of periplasmic space NiR.** The periplasmic space NiR enzyme was prepared according to a previous study [9]. Briefly, the CINR cells were suspended in enzyme extraction buffer to reach a concentration of 110 mg/ml (wet bacteria weight/buffer volume). Then, 20.00 mg/ml lysozyme was added to reach a concentration of 0.10% (mass/volume) and completely mixed. The digestion lasted for 1 h at 30°C to fully break cell walls and produce a crude periplasmic space enzyme solution. The extracts were then centrifuged at 4°C and 8000 r/min for 30 min. The supernatant was CINR periplasmic space enzyme solution and the precipitation was CINR spheroplasts.
3. **Preparation of cytoplasmic NiR.** Preparation of cytoplasmic enzymes was conducted according to a previous report [4]. The CINR spheroplasts produced in the last step were suspended in 5 fold volumes of enzyme extract buffer. The cells were sonicated for 5 min on ice, pausing every 2 s for 2 s. The sonicated cells were then centrifuged at 4°C and 8000 r/min for 30 mins. The supernatant contained CINR cytoplasmic enzyme. The cell debris pellet was resuspended in 3 ml enzyme extraction buffer. 100.00 mg/L NaNO_2_ was added to 1 ml cell debris suspension to reach a concentration of 10.00 mg/L and kept for 24 h at 30°C. The nitrite concentration was then measured as mentioned above and recorded as CK3.
4. **Nitrite degradation mediated by the induction medium without LCR 6013.** One milliliter CBINR was centrifuged for 10 min at 10000 r/min and the supernant was then filtrated with a sterilized 0.22 µm membrane filter to remove LCR 6013 cells to generate induction medium without LCR 6013. Next, 100.00 mg/L NaNO_2_ was added to the induction medium to reach a final concentration of 10.00 mg/L and kept for 24 h at 30°C to measure the concentration of nitrites and noted as CK4.
5. **Nitrite degradation without NiR.** One milliliter of CBINR was sterilized for 15 min under the conditions of 0.1 Mpa, in order to inactivate the enzyme. Then, 100.00 mg/L NaNO_2_ was added to the induction medium to reach a final concentration of 10.00 mg/L and kept for 24 h at 30°C, in order to measure the concentration of nitrites and noted as CK5.
6. **Measurement of NiR enzyme concentration.** The NiR enzyme concentration was measured using a UV spectrophotometer (Shanghai Lingguang Technology Co. Shanghai, China). Bovine serum albumin was used as the standard. The absorbance values of different concentrations of bovine serum albumin were measured at 280 nm to generate the standard curve. And the NiR enzyme concentration can be calculated based on the standard curve equation Y  = 0.618X−2.133, where Y is the absorbance × 1000, X is the protein concentration (µg/ml), and the regression coefficient R2 = 0.9996.
7. **Measurement of NiR enzyme activity.** One milligram crude enzyme protein and 100 µl of 100 mg/LNaNO_2_ were added to a 1.5 ml centrifuge tube. The final volume was adjusted to 1 ml using PBS buffer. The solution was kept for 24 h at 30°C and the nitrite concentration was measured as mentioned above. Three independent experiments were conducted. One unit of NiR enzyme activity (1 U) is defined as the amount of sodium nitrite (ng) generated by 1 mg NiR enzyme for 1 h.

### Data Analysis

The experimental data were analyzed and figures were made using Microsoft Excel 2003 software.

## Results and Discussion

### Dynamic changes of the Nitrite Concentration in Vegetable Fermentation

**Figure 1:**
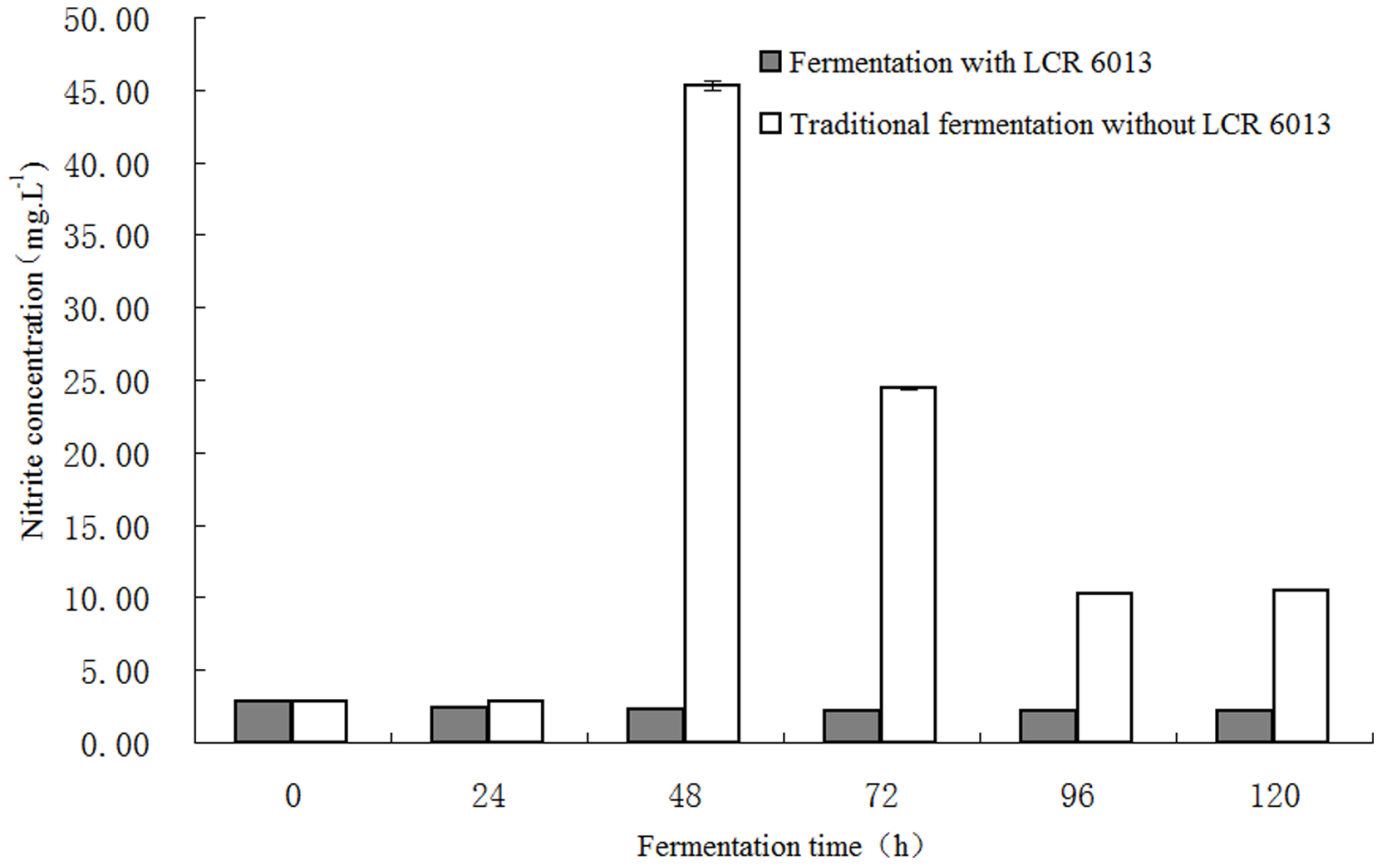
Dynamic changes of the nitrite concentration during pickle fermentation using *Lactobacillus casei* subsp. *rhamnosus* LCR 6013. After fermentation for 120°C, the nitrite concentration in the fermentation system with strain LCR 6013 was 2.21 mg/L, which was significantly lower than that in the control sample (10.50 mg/L) without the LCR 6013 strain.

The nitrite concentrations in fermented vegetables after different lengths of fermentation are shown in Figure 1. The nitrites in fresh mustard were 2.87 mg/L and were then reduced to 2.41 mg/L and 2.32 mg/L after 24 h and 48 h fermentation with LCR 6013, respectively. The nitrite concentration was further reduced to 2.21 mg/L after 72 h of fermentation and remained stable at this level. Compared to conventional fermentation, the nitrite concentration was significantly reduced in the fermentation system with LCR 6013 (*P≤0.01*). The nitrite concentration in fermented vegetables without inoculation of LCR 6013 initially experienced an increased and then decreased, with the highest concentration reaching 45.30 mg/kg after 48 h fermentation, which is consistent with a previous report [10]. Therefore, LCR 6013 can significantly inhibit the abnormal accumulation of nitrites in vegetable fermentation. The high concentration of nitrites in foods has been a worldwide research focus in food security for a long time. Hashimoto et al. studied the potential reasons for the accumulation of nitrites in Chinese cabbage kimchi [4]. Their results suggested that nitrate accumulated during the process of bacterial fermentation and the nitrate concentration was reduced with lactic acid fermentation. High concentrations of nitrites (>100 mg/L) were maintained for a considerable length of time. The abnormal accumulation of nitrites in kimchi is associated with several factors: (1) the number of coliforms is higher than control groups, (2) the concentration of soluble nitrogen compounds is higher than control groups, and (3) the buffering capacity is higher than control groups. These results suggest that the abnormal accumulation was caused by the long-term survival of coliforms that promote nitrate reduction. *Lactobacillus delbrueckii lactis* CIDCA 133 inhibits the nitrate reductase activity of *E. coli*, which is closely related with the number of viable cells of *Lactobacillus,* rather than the low pH. This suggests that some substances were directly transferred from the Lactobacillus to *E. coli* [11].

### The Effects of NaCl and Vc on Nitrite Degradation of LCR 6013

**Figure 2:**
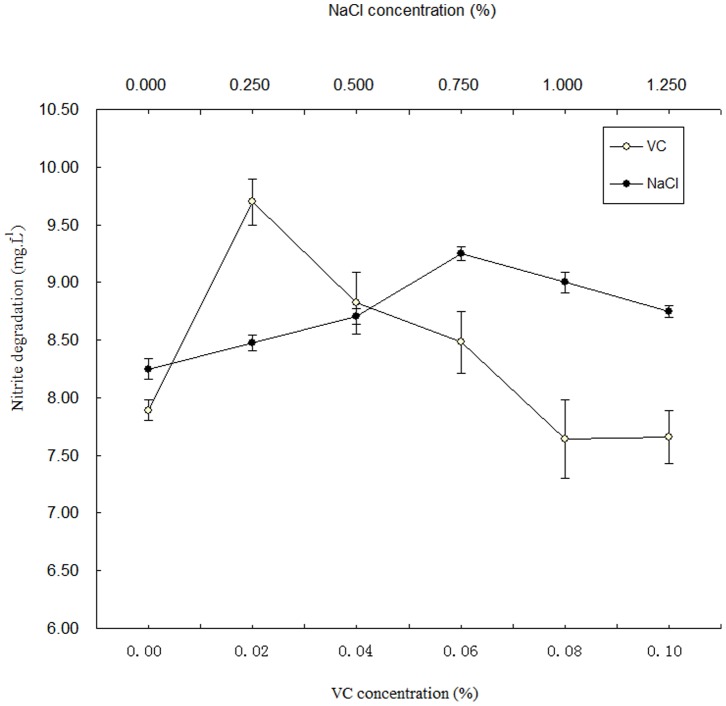
The effects of NaCl and Vc on the degradation of nitrites by *LCR* 6013 in the MRS system. The highest nitrite degradations, 9.29/L and 9.89 mg/L, were observed when NaCl and Vc concentrations were 0.75% and 0.02%, respectively, in the MRS medium.

The nitrite degradation of LCR 6013 at different concentrations of NaCl, 0.00%, 0.25%, 0.50%, 0.75%, 0.10%, 1.00%, and 1.25%, in the liquid MRS medium were compared to evaluate the effect of NaCl on the nitrite degradation of LCR 6013. The different degradation capacities are shown in Figure 2. With increased NaCl concentration, the nitrite degradation capacity increased. The nitrite degradation reached the highest value, 9.25 mg/L, when the NaCl concentration was 0.750%. After that, the nitrite degradation was reduced with increasing NaCl concentration. The nitrite degradation of *LCR 6013* was significantly higher than in the control group when the NaCl concentration was 0.750% (*p≤0.01*). The NaCl concentration generally ranges from 3.00–8.00% in vegetable fermentation [12], which was significantly higher than the concentration used in our study. The MRS medium also contains some other inorganic ions. With the addition of NaCl (>0.75%) to the MRS medium, the extremely high concentration of total inorganic ions may prevent the growth of *LCR 6013* and inhibit the activity of nitrite reductase. Given the low tolerance of LCR 6013 to salt, the concentration of NaCl used for regulating the nitrite degradation in vegetable fermentation should be kept lower to achieve the most efficient nitrite degradation.

The nitrite degradation of LCR 6013 at different initial concentrations of Vc, 0.00%, 0.02%, 0.04%, 0.06%, 0.08%, and 0.10%, in the liquid MRS medium was compared to evaluate the effect of Vc. The nitrite degradation capacities are shown in Figure 2. The NaNO_2_ degradation reached the highest value, 9.70 mg/L, when the concentration of Vc was 0.02%. However, the nitrite degradation decreased with increasing Vc, after 0.02%. LCR 6013 nitrite degradation was significantly higher than in the control group when the Vc concentration was 0.02% (*p≤0.01*). The initial Vc concentration was 0.014% and decreased with the extension of vegetable fermentation. Based on the comparison of different formulas of fermented vegetables, it has been shown that Vc reduction was consistent with nitrite production; slow production of nitrites is matched with slow Vc loss. Thus, it is speculated that Vc could be used by *Lactobacillus* after permeating pickle juice [13]. Under anaerobic conditions, when the electron donor generated by the nitrite reductase was ascorbate, N_2_O rather than No was produced by *Paracoccus halodenitrifican*s [14]. Therefore, apporpriate Vc concentration benefits nitrite degradation by LCR 6013, however, extremely high Vc concentration inhibits nitrite degradation.

### The Pathway of Nitrite Degradation of LCR 6013 in the MRS Medium

**Figure 3:**
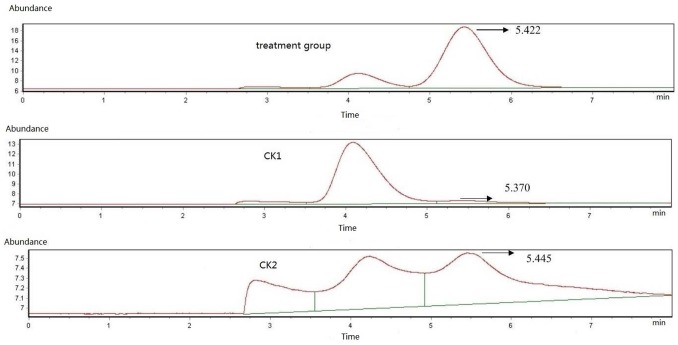
Measurement of N_2_O generated by LCR 6013 during nitrite degradation using ECD-gas chromatography. The treatment group was the experimental group. The CK1 and CK2 groups were controls without the inoculation of LCR 6013 and without the addition of 10.00/LNaNO2, respectively. The retention time of N2O was 5.422 min.

Theoretically, nitrite degradation is involved in two pathways. In the nitrate respiration pathway (NO_2_ -> NO -> N_2_O -> N_2_), cytochrome cd_1_ or copper-containing NiR serve as the nitrite reductase; nitric oxide reductase (Nor) and nitrous oxide reductase (Nos) are also involved [15]. In the ammonium formation pathway (also described as dissimilatory nitrate reduction to ammonium, DNRA pathway), the nitrite reductase is cytochrome c NrfA [16]. The NrfA in *Escherichia coli* can also convert potential intermediate products, NO and NH_2_OH, to NH_4_^+^, playing a role in NO detoxification in the intestine [17]. In the 1980s, the NO^−^_2_ degradation pathway was reported in nitrifying bacteria, but the nitrite degradation pathway of the *Lactobacillus* genus is still unclear. In this study, we conducted further experiments in order to understand which pathway contributes to the nitrite degradation by LCR 6013. ECD- gas chromatography was used to measure N_2_O in the headspace gas in the MRS reaction system. The results are shown in Figure 3. The retention time of N_2_O was 5.42 min, and the N_2_O content was 28.81×10^−6^. No N_2_O was detected in the control CK1 group (without inoculation of LCR 6013) or the CK3 control group (without addition of NaNO_2_). Therefore, N_2_O is the key indicator of nitrite degradation by LCR 6013 in the MRS reaction system.

To further confirm that the nitrate respiration pathway is involved in nitrite degradation by LCR 6013, modified indophenol blue staining was used to detect NH_4_^+^. We found that the TAN concentration in the experimental, CK1 and CK2 groups was close to 0.650 mg/L in the MRS reaction system. These results confirmed that the nitrate respiration pathway, rather than the ammonium formation pathway, contributes to the nitrite degradation of LCR 6013. The results below about the effects of different fermentation times on nitrite degradation also support this conclusion.

**Figure 4:**
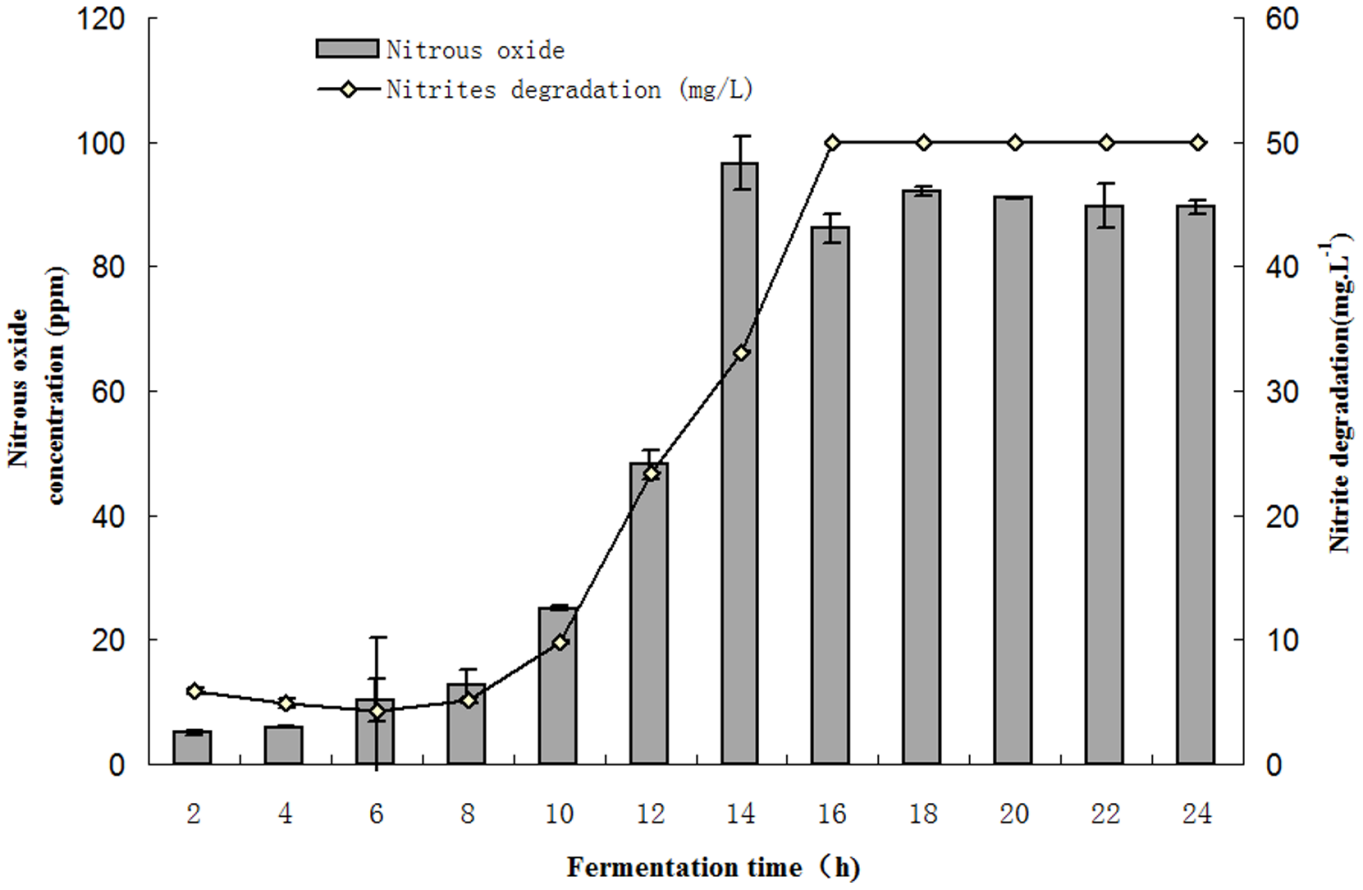
Dynamic changes of the concentration of N_2_O and nitrite in MRS fermentation system with LCR 6013.

The influence of different fermentation times on nitrite degradation and on the generation of N_2_O in LCR 6013 is shown in Figure 4. Nitrite degradation increased with the extension of fermentation time in the MRS medium when the initial NaNO_2_ concentration was 50 mg/L. After a 16-hour fermentation, the NaNO_2_ was completely degraded. With extension of the fermentation time, the N_2_O concentration increased gradually. During the 12–14 h of fermentation, the N_2_O concentration increased most rapidly and N_2_O reached the highest production, at 86.10×10^−6^–91.04×10^−6^. Thus, the degradation products of NaNO_2_ contain N_2_O. After 14 h of fermentation, the N_2_O concentration decreased slightly, probably caused by N_2_O leaking with the extension of fermentation time, or by other reactions in which N_2_O was involved. Whether and how N_2_O is involved in other reactions needs to be studied in the future.

### Subcellular Location of Nitrite Reductase (NiR)

Immunochemical assays, proton migration assays, and analyses of cell components are commonly used in studying the subcellular localization of nitrite reductase. In the cell component analysis method, the cells are treated with lysozyme to obtain periplasmic space enzymes. After breaking cells, ultracentrifugation and chapso detergents are used to obtain membrane-bound enzymes. With the development of immunochemistry, the immune labeling methods were widely used to study subcellular location of these enzymes, due to high accuracy and direct enzyme demonstration. Using the colloidal gold labeling method, Mark *et al*. [18] found NiR in *Pseudomonas aeruginosa*, an important denitrifying bacterium in the environment, located in the periplasmic space or inner membrane. However, NiRs in these two subcellular places have different characteristics and their purification methods are different. The subcellular localization of NiR in *Lactobacillus* is still unclear. In this study, the cell component analysis method was utilized to localize the NiR in LCR 6013. The bacterial cell wall was broken down by lysozyme to obtain periplasmic space enzymes and spheroplasts were broken by sonication. NiR was obtained following centrifugation. The results are shown in Table 1. No nitrite was degraded in groups CK4 and CK5, thus, no NiR was found in the supernatant. The crude enzyme in the periplasmic space and cytoplasmic enzyme of CINR exhibited nitrite degradation activity (312.64 U and 124.69 U, respectively), by NaNO_2_ degradation of 7.51 mg/L and 3.00 mg/L, respectively. The NiR enzyme activity in the periplasmic space was 2.5 times higher than that in the cytoplasm. Thus, the NiR enzyme was mainly located in the periplasmic space in *LCR 6013,* although a small amount of enzyme was located in the cytoplasm, based on the observation that cell debris (CK3 group) had nitrite degradation activity. The enzyme activities, however, were not measurable, because of the turbidity of the cell debris.

**Table 1:** The location of nitrite reductase in *LCR* 6013.

	Periplasmic NiR	Cytoplasmic NiR	CK3	CK4	CK5
NaNO_2_ degradation(mg/L)	7.51±0.038	3.00±0.091	3.43±0.02	0.013±0.002	0.017±0.003
Enzyme activity(U)	312.64±1.57	124.69±3.81	**–**	**–**	**–**

Data were presented as the mean ± standard deviation (S.D.).

## Conclusion and Perspectives

In the MRS medium, LCR 6013 reached the highest levels of nitrite degradation, 9.29 mg/L and 9.89 mg/L, when NaCl and Vc concentrations were 0.75% and 0.02%, respectively, and the initial NaNO_2_ was 10.00 mg/L. Compared to the control groups, LCR 6013 exhibited significantly more nitrite degradation (*p≤0.01*). When the initial NaNO_2_ was 10.00 mg/L, 28.81×10^−6^ N_2_O was detected in the headspace but no NH_4_^+^ was detected, thus, nitrate respiration, rather than ammonium formation, was likely involved in the nitrite degradation in LCR 6013. After 16 h of fermentation, LCR 6013 completely degraded NaNO_2_ (50.00 mg/L). There was 96.61×10^−6^ N_2_O after 14 h of fermentation when the initial NaNO_2_ was 50.00 mg/L. The NiR enzyme activity in the periplasmic space was 2.5 times that in the plasma. Based on these observations, we concluded that LCR 6013 is capable of efficiently degrading nitrites by NiR and of producing N_2_O via the nitrate respiration pathway. However, the detailed mechanisms and pathway involved in nitrite degradation in *Lactobacillus* should be further investigated. In addition, the accurate localization of NiR must be detailed by studies, for instance, with NiR antibodies. It would also be interesting and relevant to ascertain whether or not the NiR in the periplasmic space and in the plasma is the same.
